# Orthogonal halogen and hydrogen bonds involving a peptide bond model†
†Electronic supplementary information (ESI) available: Experimental part, DSC, IR spectroscopic and crystallographic data. CCDC 899779–899785. For ESI and crystallographic data in CIF or other electronic format see DOI: 10.1039/c4ce01514b



**DOI:** 10.1039/c4ce01514b

**Published:** 2014-07-31

**Authors:** Vera Vasylyeva, Susanta K. Nayak, Giancarlo Terraneo, Gabriella Cavallo, Pierangelo Metrangolo, Giuseppe Resnati

**Affiliations:** a NFMLab , D.C.M.I.C. “Giulio Natta” , Politecnico di Milano , Via Mancinelli 7 , 20131 Milan , Italy . Email: pierangelo.metrangolo@polimi.it ; Email: giancarlo.terraneo@polimi.it ; Fax: (+39) 02 2399 3180 ; Tel: (+39) 02 2399 3041; b Center for Nano Science and Technology@Polimi , Istituto Italiano di Tecnologia , Via Pascoli 70/3 , 20133 Milan , Italy; c VTT-Technical Research Centre of Finland , P.O. Box 1000 , FI-02044 VTT , Finland

## Abstract

The peptide bond model *N*-methylacetamide self-assembles with a range of dihalotetrafluorobenzenes forming co-crystals that all show the occurrence of orthogonal hydrogen and halogen bonds.

## Abstract 2

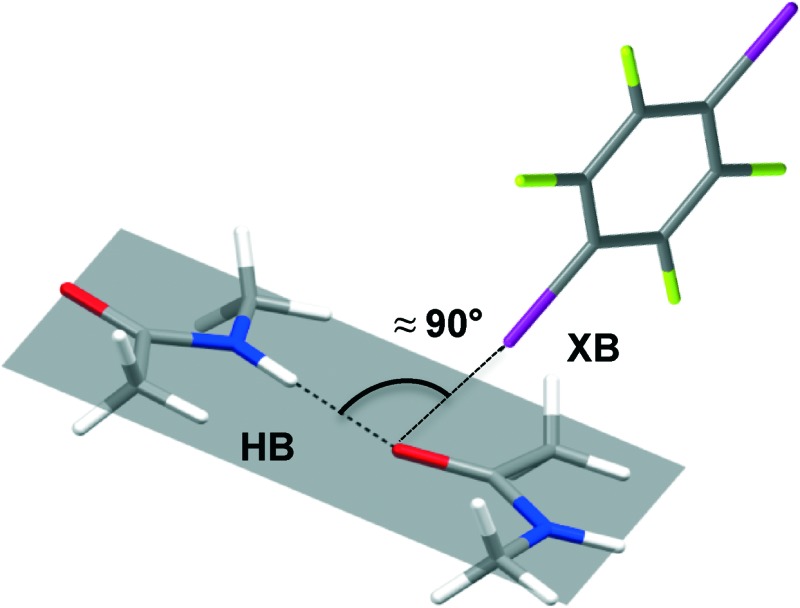
 *N*-Methylacetamide, a well-known peptide bond model, and dihalotetrafluorobenzenes form co-crystals and show geometrically orthogonal hydrogen and halogen bonds sharing the same carbonyl oxygen atom.

Orthogonal self-assembly relies on the use of multiple interaction motifs applied in the same system to drive the assembly of different components. The specific and highly controllable interactions that are used do not influence each other's assembly profile and can be manipulated independently and simultaneously.^1^

In 2009, P. S. Ho and co-workers^2^ demonstrated, through a Protein Data Bank (PDB) survey, that in protein–ligand complexes hydrogen (HB) and halogen (XB) bonds^3^ occur orthogonally, in terms of both their geometric features and their chemical behaviour, when sharing the carbonyl oxygen atom of the peptide bond as a common bond acceptor. Lately, we have demonstrated that HB and XB can successfully be combined in an orthogonal manner to drive the self-assembly of complex and functional supramolecular networks.^4^ In this latter case, the two interactions did not share the same acceptor partner. Very recently, Bruce *et al.* successfully reproduced Ho's orthogonal motifs by using *N*-methylacetamide and *N*-methylbenzamide as peptide bond models with a selected number of iodinated XB donors.^5^ However, in their hands *N*-methylacetamide decomposed to methylacetamide and any attempt to get analogous motifs involving brominated donors failed.

In this communication, we demonstrate in small-molecule self-assembly that HB and XB occur orthogonally on the same acceptor site in a very reliable and consistent manner also when brominated XB donors are used. We chose *N*-methylacetamide (NMA, **1**) as the smallest molecule that mimics the peptide bond (–NH–C=O) and thus the protein backbone.^6^ NMA effectively self-assembles with a range of dihalotetrafluorobenzenes resulting in co-crystals characterized by XBs occurring orthogonal to the classical HB pattern that characterizes the homomeric assembly of NMA.

Very few examples of ‘engineered’ orthogonal XB and HB in the context of crystal engineering can be found in the Cambridge Structural Database (CSD),^7^ one exception being the isonicotinamide–chloroacetic acid co-crystal^8^ (details about the CSD search are given in the ESI†).

Our goal was to rationally design systems based on the orthogonal HB/XB supramolecular synthon and verify its robustness for use in crystal engineering. For this reason, NMA **1**, affording the carbonyl oxygen acceptor site, was co-crystallized with several dihalotetrafluorobenzenes (DXTFBs), the XB donors, 1,2-dibromotetrafluorobenzene (12DBrTFB, **2a**), 1,3-dibromotetrafluorobenzene (13DBrTFB, **2b**), 1,4-dibromotetrafluorobenzene (14DBrTFB, **2c**), 1,2-diiodotetrafluorobenzene (12DITFB, **2d**), 1,3-diiodotetrafluorobenzene (13DITFB, **2e**), and 1,4-diiodotetrafluorobenzene (14DITFB, **2f**). The corresponding complexes **3a**–**f** were obtained (Scheme 1).

**Scheme 1:**
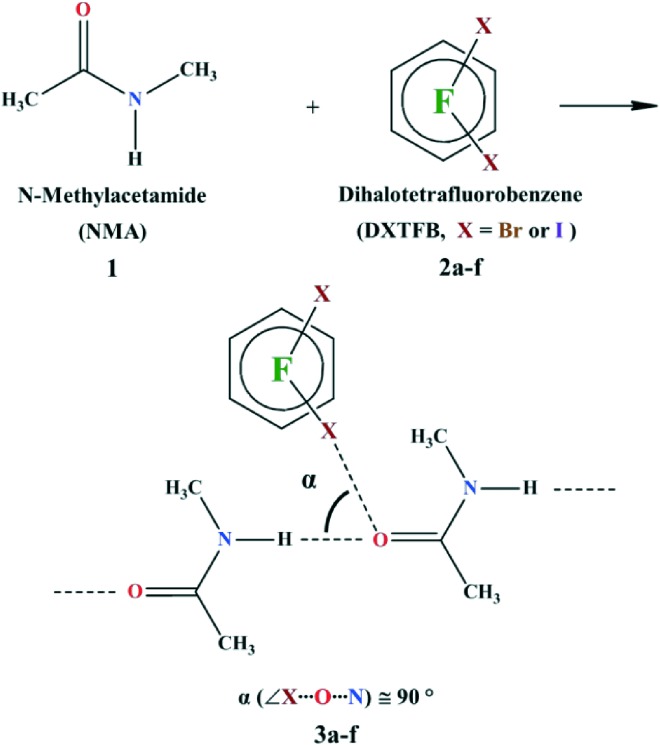
Synthesis of *N*-methylacetamide (NMA, **1**) co-crystals with various dihalotetrafluorobenzenes (DXTFB, X = Br or I). The orthogonal angle (*α*) is defined as the angle between XB (C–X···O, X = Br or I) and HB (N–H···O), *i.e.*, ∠X···O···N.

We first re-determined the crystal structure of **1** at low temperature by *in situ* cryo-crystallization using a zone-melting procedure with an optical heating and crystallization device (OHCD).^9^ This was intended to get rid of static disorder that is found in the previously reported structures of NMA.^10^ A fully ordered crystal structure of **1** resulted in the *Pnma* space group. Classical N–H···O (1.878(2) Å)^11^ HBs link NMA molecules into infinite 1D chains running along the *a* crystallographic axis (Fig. 1). The two interacting groups N–H and C=O are in the *trans* conformation, mimicking the molecular arrangement of the polypeptide backbone observed in β-sheet structures.^12^

**Fig. 1:**
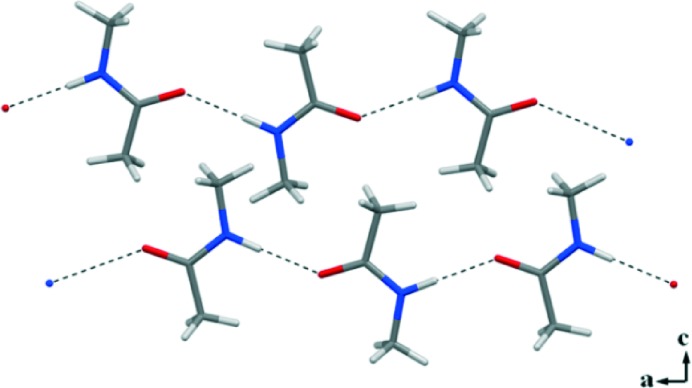
N–H···O HBs drive the formation of 1D infinite chains in the crystal packing of the homocrystal **1**.

DSC thermograms of 1 : 1 mixtures of **1** and **2a**–**f** revealed that both on cooling and on heating, the peaks for crystallization/melting of the starting compounds were not observed, confirming quantitative co-crystal formations with the adopted tectons' ratio. New melting endotherms appeared at temperatures higher than the melting point of pure NMA (12–20 °C), except for **3a** that melted at 18 °C, giving a quite sharp peak (Table 1). Interestingly, the melting points of **3a**, **b**, **d**, and **e** are higher than, and of **3c** and **f** lower than, those of the corresponding XB donor tectons.‡
‡Single-crystal X-ray diffraction data for **1** and **3a**–**f** were recorded using Mo-Kα radiation on a Bruker KAPPA APEX II diffractometer. Data were collected in *ω* and *φ* scan with a scan width of 0.5. The data were reduced with empirical absorption corrections. Structures were solved by direct methods using SHELXL97.^19^ The molecular diagrams shown were generated using Mercury 3.3.^20^ The non-hydrogen atoms are refined anisotropically and hydrogen atoms were positioned geometrically for **3a**–**d**. All crystallographic details are listed in Table S2 and intermolecular interactions are listed in Table S3 in the ESI.†



**Table 1:** IR shifts of C=O and N–H stretching modes, melting points, and orthogonal angle *α* (∠X···O···N, with X = Br, I) of **1** and co-crystals **3a**–**f**

	*ν*_C=O_ (cm^–1^)	*ν*_N–H_ (cm^–1^)	m.p. (°C)	*α* (∠X···O···N) (°)
**1**	1634	3290	12–20	—
**3a**	1635	3292	18	98.5(3)
**3b**	1629	3289	35	91.9(7)
**3c**	1625	3291	75	81.2(9)
**3d**	1616	3281	67	82.9(3); 89.0(2)^*a*^
**3e**	1617	3282	76	81.9(4)
**3f**	1607	3300	87	77.9(5)

^*a*^Two symmetry-independent molecules.

Good-quality single crystals **3a**–**f** were reproducibly obtained from 1 : 1 ratios of the starting compounds and successfully analysed by X-ray diffraction (**3a** and **3b** are low-melting solids and required the OHCD method). All adducts show remarkable similarities in their supramolecular arrangements. The classical N–H···O HBs observed in the crystal structure of pure NMA are preserved in co-crystals **3a**–**f** and organize the NMA molecules into infinite 1D chains as in pure **1**. N–H···O contacts span the range 1.831(4)–2.182(1) Å that do not differ much from the values seen in pure NMA, although in all of the adducts the NMA molecule is disordered. Moreover, in all of the structures **3a**–**f** the carbonyl O atom is involved in an additional XB involving at least one of the two XB donor sites of the dihalotetrafluorobenzenes used.

A distinctive feature of the XB is its directionality. As expected, it has been observed in the C–X···O angles of **3a**–**f**, which span the range 168.5°–177.7°. X···O interaction distances are in the range 2.800(2)–2.871(4) Å for X = Br (**3a**–**c**) and 2.706(2)–3.001(1) Å for X = I (**3d**–**f**), which correlate well to the calculated interaction energy curves of different complexes between halophenyl derivatives and NMA.^13^

Only one of the XB donor sites of the DXTFBs in **3a**–**e** is involved in the XB with the carbonyl oxygen atom. Differently, in **3f**, both of the iodine atoms of 14DITFB are halogen-bonded to the carbonyl oxygens of two NMA molecules belonging to two different hydrogen-bonded chains. In all of the six co-crystals, the oxygen atom of NMA is simultaneously involved in short HBs. Special attention during the analysis of the crystal structures has been given to the angle *α*, which has been defined as the angle between XB and HB, ∠X–O–N as shown in Scheme 1. These angles in **3a**–**f** vary in the range 77.9–98.5°, thus demonstrating the intrinsic tendency of HB and XB to occur orthogonal to each other when sharing the same sp^2^ O atom (Fig. 2).

**Fig. 2:**
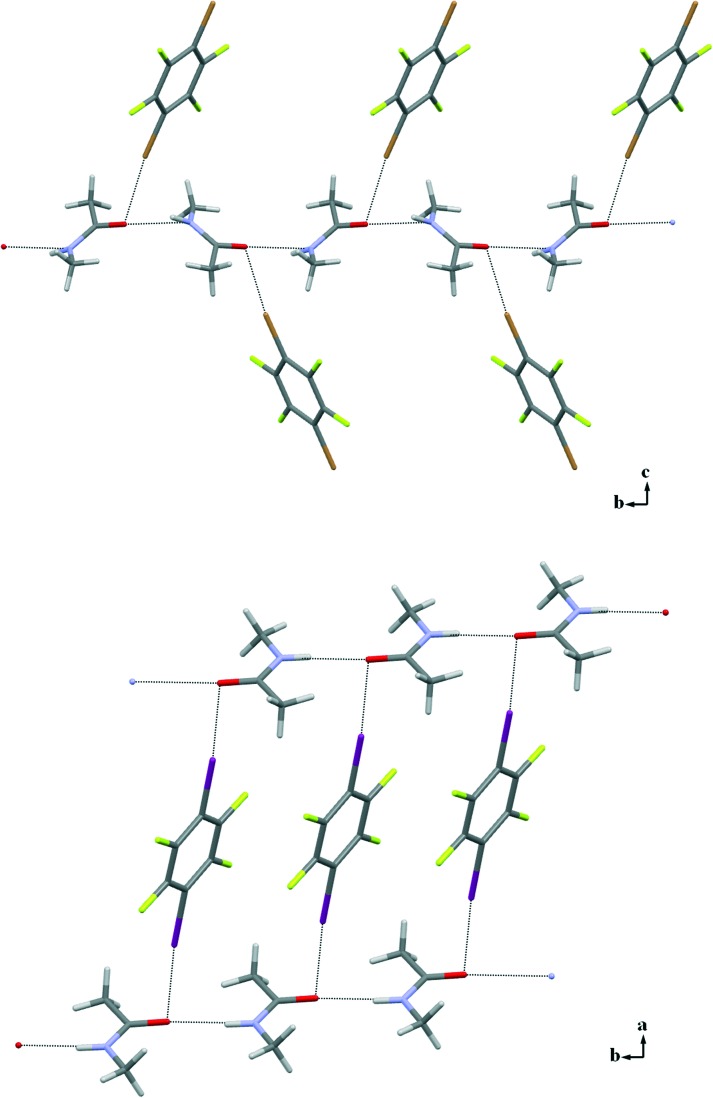
Partial crystal packing showing how infinite 1D chains (horizontally positioned) formed by NMA *via* N–H···O HBs further interact with 14DITFB *via* Br···O XBs in **3c** (top) and I···O XBs in **3f** (bottom). The disorder on the NMA molecule is omitted for clarity.

Interestingly, while in **1** and **3f** the N–H···O HB occurs perfectly in the carbonyl plane, in the structures of **3a**–**e** it assumes an out-of-plane arrangement deviating from the carbonyl plane by angles in the range 36.19°–72.57°. The orthogonal XB occurs in an out-of-plane arrangement in all of the structures **3a**–**f**, with the largest deviations in **3c** and **3f** where the positive *σ*-holes^13^ of bromine and iodine atoms enter the oxygen atom approximately in the equatorial region; the corresponding angles are 62.91° in **3c** and 99.41° in **3f** (see the ESI,† Fig. S4). Similar out-of-plane X···O interactions have been recently noted in protein–ligand complexes.^14^

The IR spectra of the co-crystals **3a**–**f** essentially contain modified vibrations of both the corresponding starting compounds and the observed band shifts may give an indication of how much the orthogonal HB and XB perturb the electron density of the carbonyl group. Pure NMA shows a broad *ν*_C=O_ centred at 1634 cm^–1^. This band is consistently red-shifted in all of the co-crystals **3a**–**f**, the largest shift (27 cm^–1^) being observed in **3f**. These red shifts suggest that the simultaneous formation of HB and XB results in a reduced electron density on the carbonyl group with respect to the pure NMA.^15^ Interestingly, iodinated co-crystals show larger shifts than brominated co-crystals, and *para* derivatives show larger shifts than *ortho* and *meta* derivatives. The shifts of the N–H stretching modes of the amide group (at 3290 cm^–1^ in pure **1**) do not show any clear trend. Moreover, no correlation has been found between the N–H stretching values relative to the increasing energies of the XBs, as reflected in C=O stretching, demonstrating that the interactions are truly independent (see plot in the ESI†).

In conclusion, we have reported the first halogen-bonded co-crystals formed by differently substituted (*o*, *m*, and *p*) dihalotetrafluorobenzenes (halogen = I and Br), functioning as halogen-bond donor modules, and *N*-methylacetamide, a well-known peptide bond model. Six co-crystals were obtained and they all show geometrically orthogonal hydrogen and halogen bonds involving simultaneously the carbonyl oxygen atom. This demonstrates the great robustness of this orthogonal synthon, which occurs in 100% supramolecular yield^16^ of the attempted co-crystals. The two interactions are also chemically orthogonal as the XB formation does not alter the 1D and hydrogen-bonded β-sheet mimetic chains typical of the homomeric assembly of *N*-methylacetamide. A PDB survey performed by Ho *et al.* showed that hydrogen and halogen bonds are orthogonal, both in terms of their geometric alignments and their chemical behaviour, when sharing the carbonyl oxygen atom of a peptide bond. Our results demonstrate that this feature is not a peculiarity occurring only when biomacromolecules are involved,^3^ but it is a general feature of the two interactions and is probably inherent to their intrinsic chemical nature.

The reported results pave the way to a new design concept in orthogonal self-assembly and crystal engineering and may also have important implications in other fields such as materials processing. As far as this field is concerned, amyloid and silk fibers are examples of ordered nanomaterials and they both feature robust β-sheet elements. The manipulation of the self-assembly and structural complexity of these nanomaterials during processing is still far from being completely understood.^17^ An approach based on the orthogonal interaction of β-sheets with halogen-bond donor mesogens may be particularly valuable.^18^ Current studies in our laboratory are addressing this issue and the results will be reported elsewhere.
