# Supplementary material for: Orthogonal halogen and hydrogen bonds involving a peptide bond model
Source: CrystEngComm. 2014 Jul 31;16(35):8102–5. doi: 10.1039/c4ce01514b (PMC4317971; doi:10.1039/c4ce01514b)
Supplement: Supplementary file 1 [file CE-016-C4CE01514B-s001.pdf]

This journal is © The Royal Society of Chemistry 2014

Orthogonal halogen and hydrogen bonds involving a peptide bond model

Vera Vasylyeva,[a] Susanta K. Nayak,[a,b] Giancarlo Terraneo,[a,b]* Gabriella Cavallo,[a,b] Pierangelo Metrangolo[a,b,c]* and Giuseppe Resnati[a,b]

Electronic Supplementary Information

Contents

		Page
S.1	Supramolecular synthesis	3
S.2	Crystallization procedure and crystal structure determination	3
S.3	CSD survey	4
S.4	Differential Scanning Calorimetry (DSC) data	5
S.5	Infrared (IR) spectroscopic data	6
S.6	NMR data	7
S.7	Crystallographic data of **1** and **3a-f**	8
S.8	Intermolecular interactions in **1** and **3a-f**	12
S.9	R_2C=O in- and out-of-plane interactions	13
S.10	References	15

S.1 Supramolecular synthesis

Starting materials **1** and **2a-f** were purchased from commercial suppliers (Sigma-Aldrich, Apollo Scientific, and ABCR) and used without further purification. **1** was first mixed with each of the compounds **2a-f** in a molar 1:1 ratio. Solid mixtures were then dissolved in chloroform and all samples were sonified for 10 min. In a second type of procedure the solid mixtures were heated up until complete melting, stirred for one hour, and cooled down to room temperature (RT).

S.2 Crystallization procedure and crystal structure determination

1, **3a**, and **3b** were *in situ* cryo-crystallized on the diffractometer in sealed 0.3 mm Lindemann capillaries according to the reported procedure.[1] **3c-f** were dissolved in and crystallized from chloroform by slow evaporation method at room temperature (from hours to days). Suitable crystals were chosen for the single crystal X-ray study. To check the reproducibility and to avoid any solvent interference, suitable single crystals were also obtained from their melts by slow cooling to RT, which resulted in the same crystal phases.

The crystals were measured using Mo-Kα radiation on a Bruker KAPPA APEX II diffractometer with a Bruker KRIOFLEX low temperature device. Crystal structures were solved by direct method and refined against F^2 using SHELXL97.[2] Packing diagrams were generated using the CSD software Mercury 3.3.[3] Intermolecular interactions were analyzed with PLATON.[4] The non-hydrogen atoms were refined anisotropically and hydrogen atoms were refined using difference Fourier map or positioned geometrically. The NMA molecule in all of the determined co-crystal structures is disordered, which appears mainly from a static randomness of molecular orientation.[5] For the measurement of distances and angles of intermolecular interactions in **3a-f**, the major component atoms of the disordered NMA molecules were considered. Crystal structures were deposited in the CSD database. CCDC 899779 – 899785 contain the supplementary crystallographic data for this paper. These data can be obtained free of charge from The Cambridge Crystallographic Data Centre via www.ccdc.cam.ac.uk/data_request/cif.

S.3 CSD survey

For the CSD search (ConQuest Version 1.15, 2013), following parameters were defined: Two component systems (number of chemical units = 2); oxygen atom of the $R_2C{=}O$ group shares both halogen and hydrogen bonds; intermolecular interactions are within the sum of VdW radii + 0.2 Å; angle X···O···Y (X = any halogen, Y = any atom of Y-H group). Outcome: 535 entries with a X···O···Y angle distribution in the range 26.27-176.75°

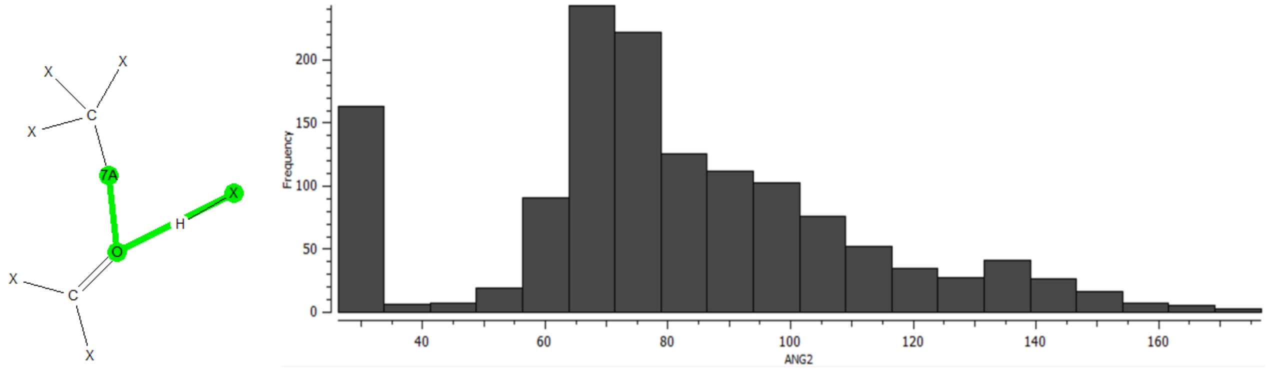

Fig. S1 CSD search: X···O···Y angle distribution within 535 entries.

S.4 Differential Scanning Calorimetry (DSC) data

DSC analyses were performed using a Mettler Toledo DSC 823e instrument. 1.5-2.5 mg of each sample were measured in a closed aluminum pan with heating/cooling rates of 10 °C/min or 5 °C/min. In the case of 12-DBrTFB, two reversible phases were observed. ^{19}F-NMR spectrum of **2a** (Fig. S2) confirmed the purity of the starting material. This thermal behavior of the sample may correspond to solid-solid phase transition between two polymorphic modifications and is not induced by impurities.

Table S1. Experimental melting points of the starting compounds **1** and **2a-f**.

Compound	1	2a	2b	2c	2d	2e	2f
m.p. (°C)	12-20	-3<br>15	6	78	53	26	107

S.5 Infrared (IR) spectroscopic data

IR spectra were obtained using a Nicolet Nexus FTIR spectrometer equipment with U-ATR device. Selected bands for the spectra of the starting compounds **1** and **2a-f**, and of the adducts **3a-f** measured at room temperature are reported. The C=O vibration of NMA is broad and span the wavenumbers 1650-1634 cm^{-1} with the sharpest position at $\nu_{C=O}$ = 1634 cm^{-1}.

1: 3290, 3102, **1634 ($\nu_{C=O}$)**, 1557, 1411, 1371, 1298, 1160, 1041, 989, 713, 597 cm^{-1}.

2a: 1613, 1499, 1459, 1352, 1273, 1117, 1034, 848, 801, 638 cm^{-1}.

2b: 1616, 1485, 1447, 1353, 1328, 1082, 894, 742, 713, 700, 639 cm^{-1}.

2c: 1480, 1448, 1359, 1294, 1233, 992, 953, 786, 775, 582 cm^{-1}.

2d: 1602, 1488, 1437, 1309, 1260, 1109, 1022, 1009, 813, 771, 633, 470 cm^{-1}.

2e: 1614, 1582, 1469, 1432, 1349, 1319, 1071, 1054, 886, 868, 723, 711, 660, 638 cm^{-1}.

2f: 1456, 1426, 1355, 1214, 985, 968, 939, 758, 564 cm^{-1}.

3a: 3292, 3101, **1635 ($\nu_{C=O}$)**, 1562, 1501, 1463, 1412, 1371, 1299, 1160, 1116, 1034, 989, 849, 803, 717, 628, 597 cm^{-1}.

3b: 3289, 3107, **1629 ($\nu_{C=O}$)**, 1569, 1487, 1446, 1373, 1308, 1159, 1071, 991, 894, 741, 699, 629, 600 cm^{-1}.

3c: 3291, 3113, **1625 ($\nu_{C=O}$)**, 1568, 1475, 1455, 1411, 1372, 1304, 1161, 1044, 987, 950, 791, 602, 588 cm^{-1}.

3d: 3281, 3115, **1616 ($\nu_{C=O}$)**, 1572, 1484, 1437, 1371, 1306, 1158, 1102, 1022, 816, 774, 752, 606, 438 cm^{-1}.

3e: 3282, 3112, **1617 ($\nu_{C=O}$)**, 1569, 1470, 1431, 1373, 1311, 1159, 1067, 1051, 995, 886, 869, 722, 708, 659, 598, 443 cm^{-1}.

3f: 3300, 3118, **1607 ($\nu_{C=O}$)**, 1571, 1456, 1408, 1371, 1307, 1217, 1160, 1040, 984, 972, 940, 760, 626, 602, 571 cm^{-1}.

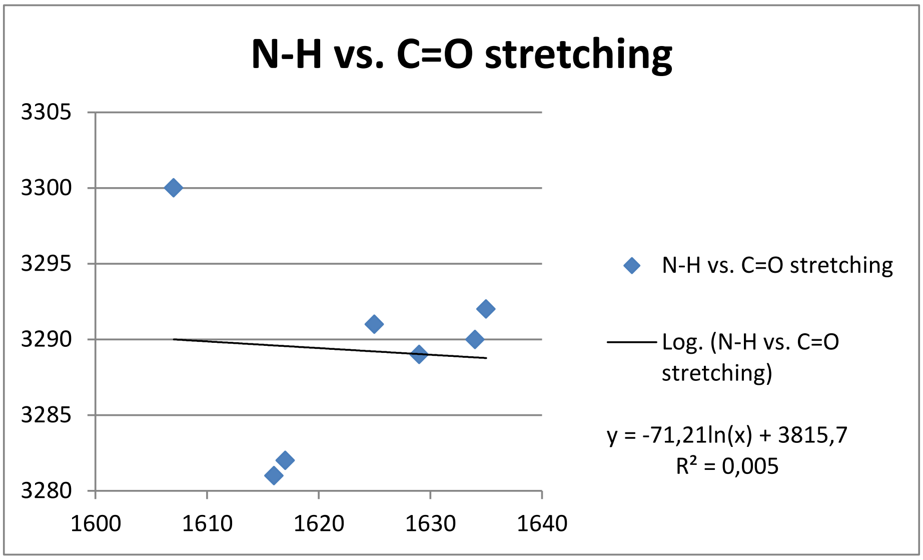

S.6 NMR data

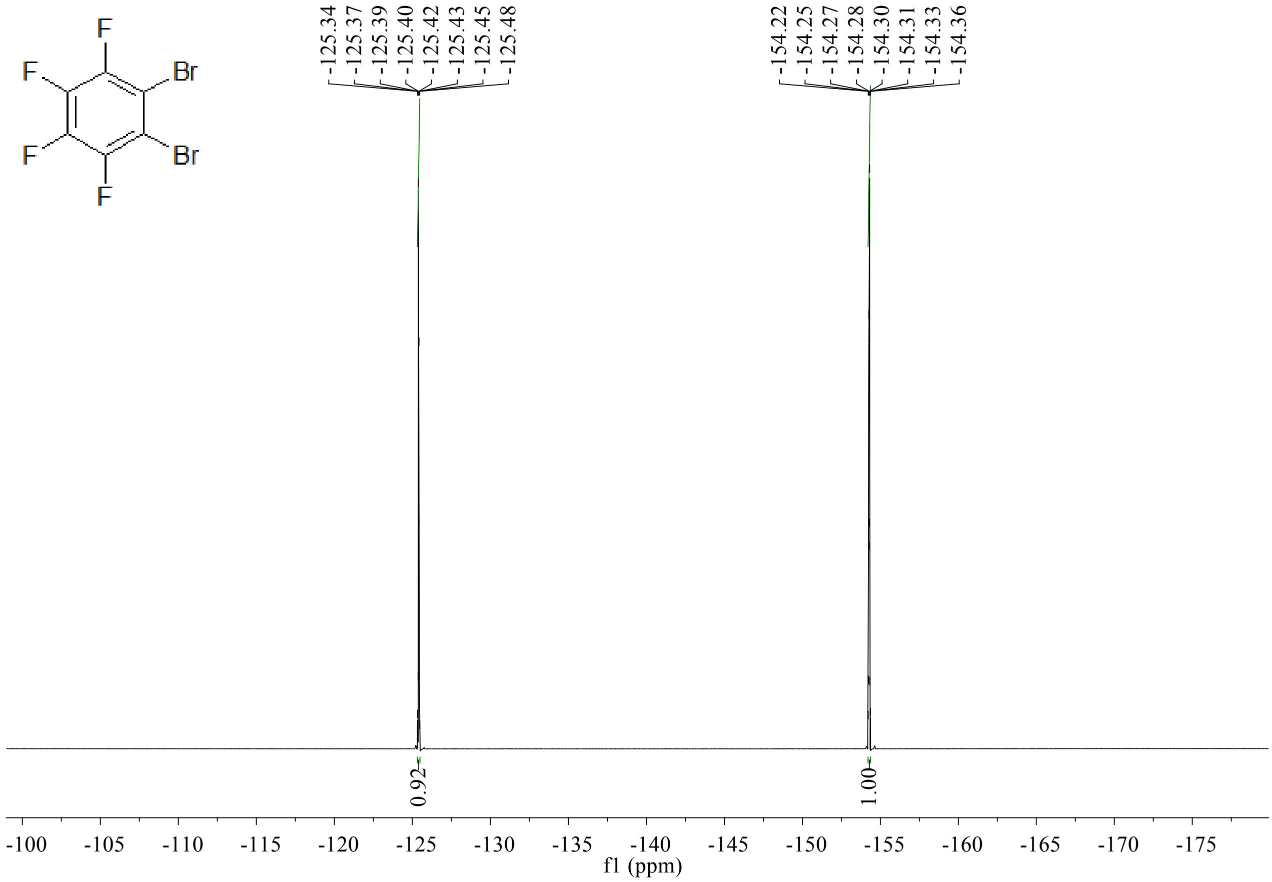

Fig. S2 ^{19}F-NMR spectrum of **2a** in $CDCl_3$.

S.7 Crystal structures of 1 and 3a-f

Table S2 Selected crystallographic and refinement parameters of crystal structures of **1** and **3a-f**.

	**3a**	**3b**	**3c**	**3d**	**3e**	**3f**	**1**
**Formula**	$(C_6F_4Br_2)$ (C_3H_7NO)	$(C_6F_4Br_2)$ (C_3H_7NO)	$(C_6F_4Br_2)$ (C_3H_7NO)	$(C_6F_4I_2)$ (C_3H_7NO)	$(C_6F_4I_2)$ (C_3H_7NO)	$(C_6F_4I_2)$ (C_3H_7NO)	C_3H_7NO
**Formula weight**	381.0	381.0	381.0	475.0	475.0	475.0	73.1
**Temperature K**	190 (2)	100(2)	100(2)	100(2)	100(2)	100(2)	100(2)
**Space group**	$P\,2_1/c$	$P\,2_1/n$	$P\,2_1/c$	P-1	$P\,2_1/c$	$C\,2/c$	*Pmna*
$\boldsymbol{a}$ **(Å)**	7.5319(13)	7.7932(6)	5.8653(3)	7.2201(3)	8.0655(5)	21.263(9)	9.6481(12)
$\boldsymbol{b}$ **(Å)**	20.560(3)	19.6616(14)	9.0752(5)	8.6847(3)	18.2945(10)	4.7949(19)	6.3391(8)
$\boldsymbol{c}$ **(Å)**	8.9505(14)	8.8023(6)	22.3773(13)	21.5893(8)	9.1049(5)	12.348(6)	7.1825(9)
$\boldsymbol{\alpha}$ **(°)**	90	90	90	85.660(2)	90	90	90
$\boldsymbol{\beta}$ **(°)**	114.813(8)	115.553(4)	95.126(3)	81.022(2)	105.695(3)	91.64(2)	90
$\boldsymbol{\gamma}$ **(°)**	90	90	90	74.403(2)	90	90	90
**Volume (Å^3)**	1258.1(4)	1216.82(15)	1186.35(11)	1287.11(8)	1293.38(11)	1258.4(9)	439.28(1)
$\boldsymbol{Z}$	8	8	8	8	8	8	4
**Density (gcm^{-3})**	2.01	2.08	2.13	2.45	2.44	2.51	1.11
$\boldsymbol{\mu}$ **(mm^{-1})**	6.475	6.695	6.867	4.918	4.894	5.031	0.083
$\boldsymbol{F}$ **(000)**	727.9	727.9	727.9	871.9	871.9	871.9	160.0
$\boldsymbol{\theta_{min, max}}$ **(°)**	(2.0, 26.0)	(2.8, 26.4)	(1.8, 26.0)	(1.0, 26.0)	(2.6, 28.3)	(1.9, 26.0)	(3.5, 26.0)
$\boldsymbol{h_{min, max}}$	(-6, 9)	(-9, 9)	(-7, 7)	(-8, 8)	(-10, 10)	(-26, 26)	(-11, 11)
$\boldsymbol{k_{min, max}}$	(-25, 24)	(-24, 24)	(-11, 11)	(-10, 10)	(-24, 24)	(-5, 5)	(-7, 7)
$\boldsymbol{l_{min, max}}$	(-10, 10)	(-11, 11)	(-27, 27)	(-26, 26)	(-12, 11)	(-15, 15)	(-8, 8)
**No. of refl. measured**	12208	11226	23328	49634	34556	7836	8597
**No. unique refl.**	2451	2487	2323	5024	3213	1215	471
**No of parameter**	168	174	168	336	205	103	48
$\boldsymbol{R_{\_all}, R_{\_obs}}$	0.107, 0.048	0.046, 0.027	0.046, 0.036	0.037, 0.028	0.033, 0.021	0.0255, 0.019	0.054, 0.047
$\boldsymbol{wR_{2\_all}, wR_{2\_obs}}$	0.139, 0.109	0.059, 0.054	0.110, 0.095	0.081, 0.071	0.043, 0.040	0.054, 0.041	0.134, 0.130
$\boldsymbol{\Delta\rho_{max, min}}$ **(eÅ^{-3})**	0.63, -1.15	0.486, -0.396	1.79, -0.951	1.41, -0.964	0.766, -0.793	0.868,-0.644	0.751, -0.258
**G.o.F**	0.954	1.048	1.173	1.234	1.021	1.232	1.078

Adduct **3a** crystallizes in monoclinic $P2_1/c$ space group and Z = 4 (Table S2). Classical N-H···O HBs (1.955(2) Å) link NMA molecules into infinite molecular chains similar to the HBs observed in the crystal structure of pure NMA. Only one of the bromine atoms of **2a** is engaged in XB with the carbonyl O atom of NMA, with Br···O distance of 2.800(2) Å and C-Br···O angle of 177.47 °. The second bromine atom undergoes a Br···F (3.313(5) Å) short contact, which links **2a** molecules into 1D chains. The ∠Br···O···N = α is equal to 97.2 °. Additional π···π (3.862(4) Å) and C-H···F (2.543(5) Å) contacts stabilize the crystal packing characterized by alternate NMA and 12DBrTFB layers interlinked by orthogonal XB/HB synthons (Fig. S5a).

Adduct **3b** was obtained in a monoclinic $P2_1/n$ space group with four molecules in the unit cell (Table S2). The intermolecular interactions and crystal packing are similar to those of **3a** (Fig. S5b). The Br···O halogen bond (2.845(2) Å) is nearly perfectly perpendicular to the N-H···O hydrogen bond (2.016(2) Å) with an α angle of 91.9 °. Further shifted π···π (3.730(2) Å) stacking interactions together with short Br···F (3.087(1) Å) and C-H···F (2.571(5) Å) contacts additionally stabilize the packing of hydrogen and halogen-bonded infinite chains of NMA and 13DBrTFB. The similarities in the crystal packing of **3a** and **3b** suggest no relevant effect of bromine *ortho-* and *meta-*substitutions on their supramolecular assembly.

As shown in Table S2, NMA:14DBrTFB adduct **3c** crystallizes in a monoclinic $P2_1/c$ space group with Z = 4. Usual infinite hydrogen-bonded NMA chains are formed with N-H···O HBs (1.883(3) Å). Similarly orthogonal Br···O XBs (2.871(4) Å) occur nearly perfectly perpendicular to the N-

H···O HBs with an α angle of 81.2 °. The second Br atom of the 14DBrTFB molecule undergoes a short intermolecular contact with the pi-electron cloud of the carbonyl group, with a Br···carbonyl (C=O centroid) distance of 3.456(3) Å. The crystal packing is additionally stabilized by weak F···F and F···C contacts (Fig. S3).

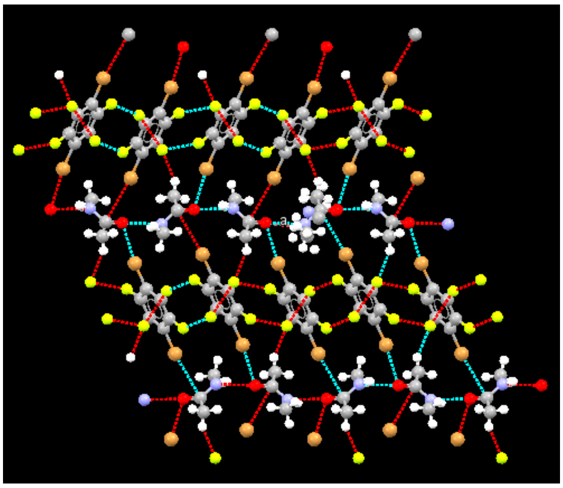

Fig. S3 Crystal lattice of **3c**.

3d crystallizes in a triclinic *P*-1 space group (Table S2) with two independent molecules of each component (NMA and 12DITFB) in the asymmetric unit. Layers of hydrogen bonded NMA chains alternate with layers containing XB donor molecules. Both independent NMA molecules (**A** and **B**) participate in formation of the same infinite N1-H···O1 hydrogen-bonded (2.010(1) Å) chains of NMA molecules ordered in a **ABABAB** arrangement (see Fig. S4). In contrast, the two independent 12DITFB molecules (**C** and **D**) contribute to the crystal architecture in different manner. In this case each layer contains only symmetry equivalent molecules **C** or **D**. Molecule **C** interacts by means of just one iodine atom under formation of short and directional I1···O1 XB (2.820(3) Å), while the other iodine is not involved in any interactions. Both iodine atoms of the second independent 12DITFB molecule are involved in intermolecular interactions, one undergoes short I4···O1 XB (2.826(2) Å) and the other forms short type II I3···I4 contacts (3.951(2) Å), connecting two neighboring **D** molecules into dimer pairs. The orthogonal XB/HB synthons occur between following pairs of symmetry independent molecules: **A/C** and **B/D** with different α angles of 82.8 ° and 88.9 °.

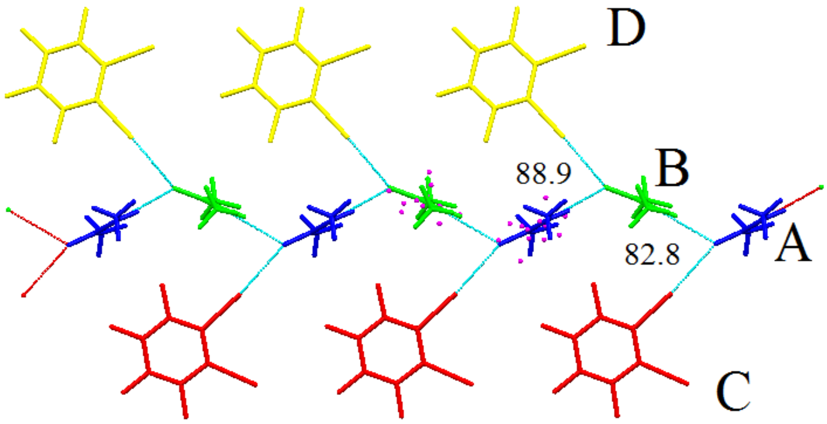

Figure S4.

Co-crystal **3e** crystallizes in a monoclinic $P2_1/c$ space group similarly to **3a-c**. Also here NMA molecules are linked into 1D chains through N-H···O HBs (2.182(4) Å) and the *meta*-substituted diiodotetrafluorobenzene participates in I···O XB (2.706(2) Å) with only one I atom while the other forms short I···F contact (3.325(2) Å). I···O XBs are at an α angle of 81.9° to N-H···O hydrogen bonds. Contrary to the previous adducts here the separation between two parallel NMA chains is extended by a second row of 13DITFB molecules linked to the first one by short F···F (2.895(2) Å) contacts. Similar to its bromine isomer (**3b**), other weak interactions such as shifted π···π (3.866(3) Å) stacking and C-H···F (2.616(3) -2.670(3) Å) contacts stabilize the crystal packing (Fig. S5d).

Crystal structure solution of the co-crystal **3f** resulted in a monoclinic $C2/c$ space group and Z = 4 (Table S2). Unlike adducts **3a-e**, the 14DITFB molecule sits on a center of inversion whereas the NMA molecule sits on a 2–fold axis with the usual static disorder.[5] The iodine atoms, where one is generated by symmetry operation, form I···O XB (3.001(1) and 3.000(3) Å) and directly interlink the infinite chains of N-H···O bonded NMA molecules. The XBs approach N-H···O HBs (2.023(3) and 1.910(2) Å) chains with an angle of 78.2 °.

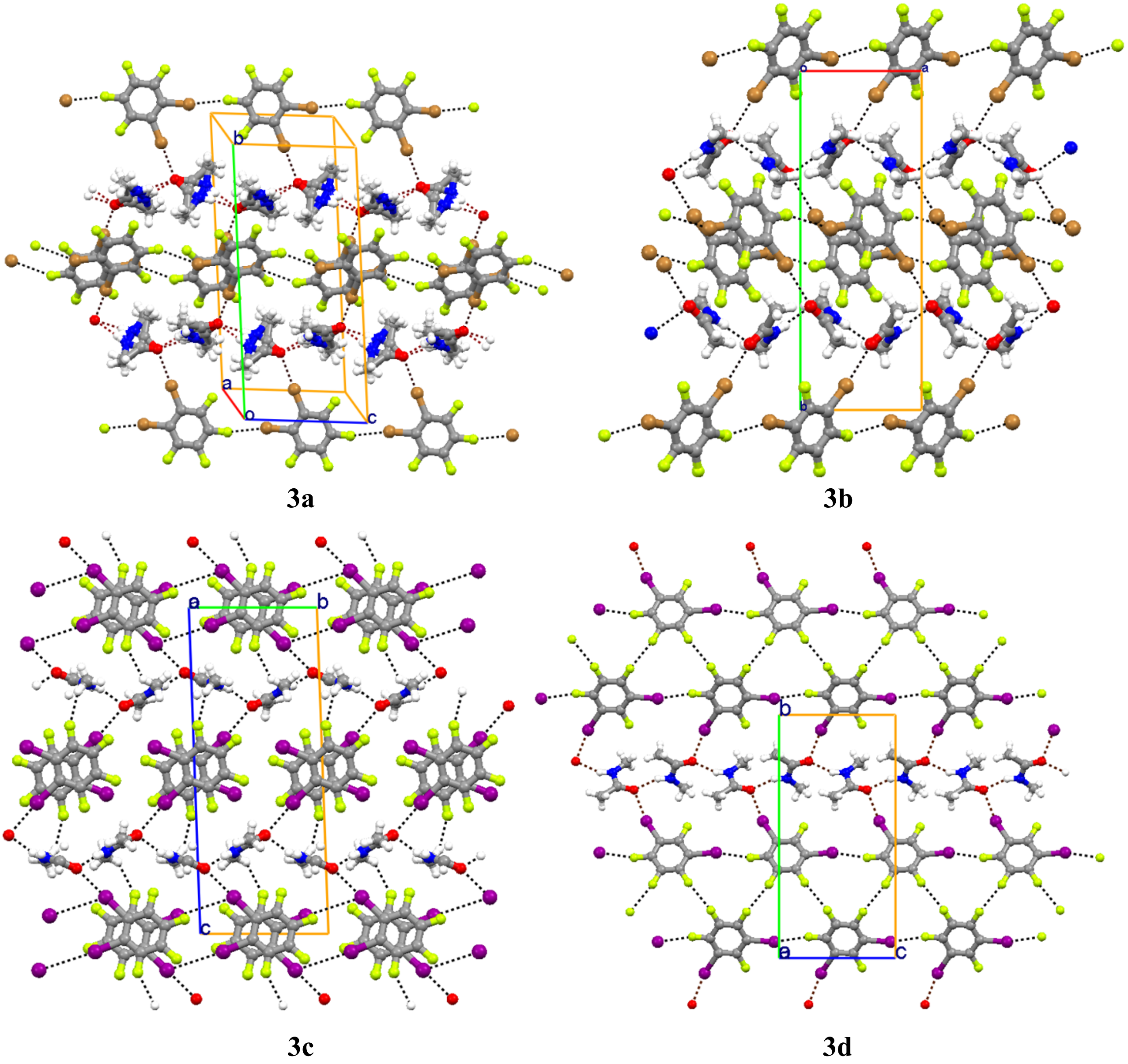

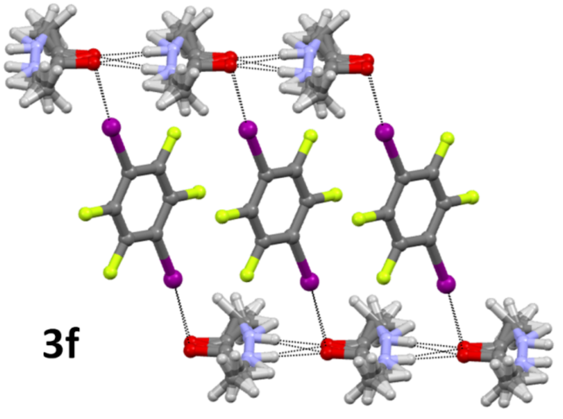

Fig. S5 Crystal lattices of **3a**, **3b**, **3d**, and **3e**. Intermolecular interactions are shown as dotted lines. In all of the structures, N-H···O HBs form infinite molecular chains, which interact with the dihalotetrafluorobenzenes by Br···O (a and b) and I···O (c and d) XBs in nearly orthogonal XB/HB fashion. Additional weak interactions stabilize the crystal packing a) Br···F, C-H···F and π···π interactions in **3a**, b) Br···F and π···π interactions in **3b**, c) I···I and C-H···F intermolecular interactions in **3d**, d) short I···F and F···F interactions in **3e**, e) the disordered NMA is shown. Color code: dark grey, carbon; green, fluorine; brown, bromine; violet, iodine; red, oxygen; blue, nitrogen; white, hydrogen.

S.8 Intermolecular interactions in 1 and 3a-f

Table S3 Intermolecular interactions in **1** and **3a-f**. Interactions involved in the formation of the orthogonal XB/HB synthon are highlited in bold.

	**C-X···Y-Z**	**X···Y/Å**	**∠C-X···Y/°**	**∠C-Y···X/°**	**Symmetry code**	**α/°∠X···O···N[b]**
**1**	N1-H1···O1	1.878(2)	178.4	-	-	
**3a**	**C1-Br1···O1A-C7A**	**2.800(2)**	**177.5**	**152.0**	**x,-y+1/2,+z-1/2**	**98.53**
	C2-Br2···F4-C5	3.313(5)	148.2	147.6	x,+y,+z-1	
	C9-H9B···F3	2.543(5)<br>3.225(7)	130.2	-	-x+1,+y-1/2,-z+1/2	
	**N1A-H1A···O1A**	**1.952(3)**<br>2.701(3)	**174.9**	**-**	**x,-y+1/2,+z-1/2**	
	Cg1···Cg1[a]	3.862(4)			2-x,-y,1-z	
**3b**	**C1-Br1···O1A-C7A**	**2.845(2)**	**168.8**	**157.9**	**x, y, z**	**91.90**
	C3-Br2···F4-C6	3.087(1)	156.1	154.3	x+1,+y,+z+1	
	C9-H9B···F3	2.571(5)<br>3.226(4)	127.9	-	-x+1/2+1,y-1/2,-z+1/2+2	
	C8-H8A···F1	2.555(2)<br>3.486(4)	152.2	-	x+1/2,-y-1/2,+z+1/2	
	**N1A-H1A···O1A**	**2.016(2)**<br>2.893(3)	**174.6**	**-**	**x+1/2,-y-1/2,+z+1/2**	
	Cg1···Cg1[a]	3.730 (2)			-x,-y,2-z	
**3c**	**C1-Br1···O1A-C7A**	**2.871(4)**	**177.7**	**117.0**	**x-1,-y+1/2+1,z-1/2**	**81.22**
	C4-Br2···C7A	3.505(3)	170.7	-	-	
	C2-F1···F2-C3	2.828(4)	157.8	138.1	-	
	C4-F3···F4-C6	2.853(5)	160.7	131.3	-x+1,-y+2,-z+2	
	**N1A-H1A···O1A**	**1.878(2)**<br>2.651(2)	**147.0**	**-**	**-x+1,+y-1/2,-z+1/2+1**	
**3d**	**C1-I1···O1C-C7C**	**2.820(3)**	**173.7**	**134.6**	**-**	**82.85**
	**C8-I4···O1A-C7A**	**2.826(2)**	**172.4**	**136.7**	**x,+y+1,+z**	**88.96**
	C7-I3···C7A	3.619(2)	122.5		x,+y+1,+z	
	C8-I4···I3-C7	3.951(2)	119.5	164.7	-x+1,-y+2,-z	
	C10-F6···F7-C11	2.866	156.7	126.4	-x+2,-y,-z	
	C5-F3···F3-C5	2.921	115.6	115.6	-x+1,-y-1,-z+1	
	C9A-H9A···F3	2.481(3)<br>3.358(1)	173.3	-	-x,-y,-z+1	
	C8D-H9c1···F3	2.540(2)<br>3.268(3)	145.0	-	-x+1,-y+1,-z	
	**N1A-H1A···O1C**	**1.831(4)**<br>2.683(4)	**162.3**	**-**	**-**	
	**N1-H1C···O1A**	**2.010(1)**<br>2.870(3)	**165.3**	**-**	**-**	
	Cg1···Cg1[a]	3.848(3)	-	-	2-x,-y,1-z	
	Cg2···Cg2[a]	3.753(2)	-	-	2-x,1-y,-z	
**3e**	**C1-I1···O1**	**2.706(2)**	**170.8**	**130.96**	**x,+y,+z+1**	**81.86**
	**N1A-H1A···O1A**	**2.182(1)**<br>2.952(6)	**142.2**	**-**	**x,-y+1/2+1,+z-1/2**	
	C3-I2···F4	3.325(2)	143.1	146.5	x,+y,+z+1	
	C9B-H9B···F2	2.616(3)<br>3.544(4)	156.8	-	-x+1,-y+1,-z+1	
	C8A-H8C···F3	2.670(3)<br>3.472(4)	151.3	-	-x,-y+1,-z	
	C4-F2···F3-C2	2.895(2)	172.7	135.6	x,-y+1/2,+z+1/2	
**3f**	**C1-I1···O1-C7**	**3.001(1)**	**172.7**	**100**	**x-1,+y-1,+z**	**77.91**
	**N1A-H1A···O1A**	**2.023(3)**	**165.0**	**-**	**x,+y-1,+z**	

[a] Cg1 (π) = Centroid of the ring C1-C6 and Cg2 (π) = Centroid of the ring C7-C12, [b] X = Br, I

S.9 $R_2C=O$ in- and out-of-plane interactions

The plane was set through four atoms of an NMA molecule: C1, C2, N1 and O1. The angle between the $R_2C=O$ plane and an involved HB or XB was measured to determine the out-of-plane deviation.

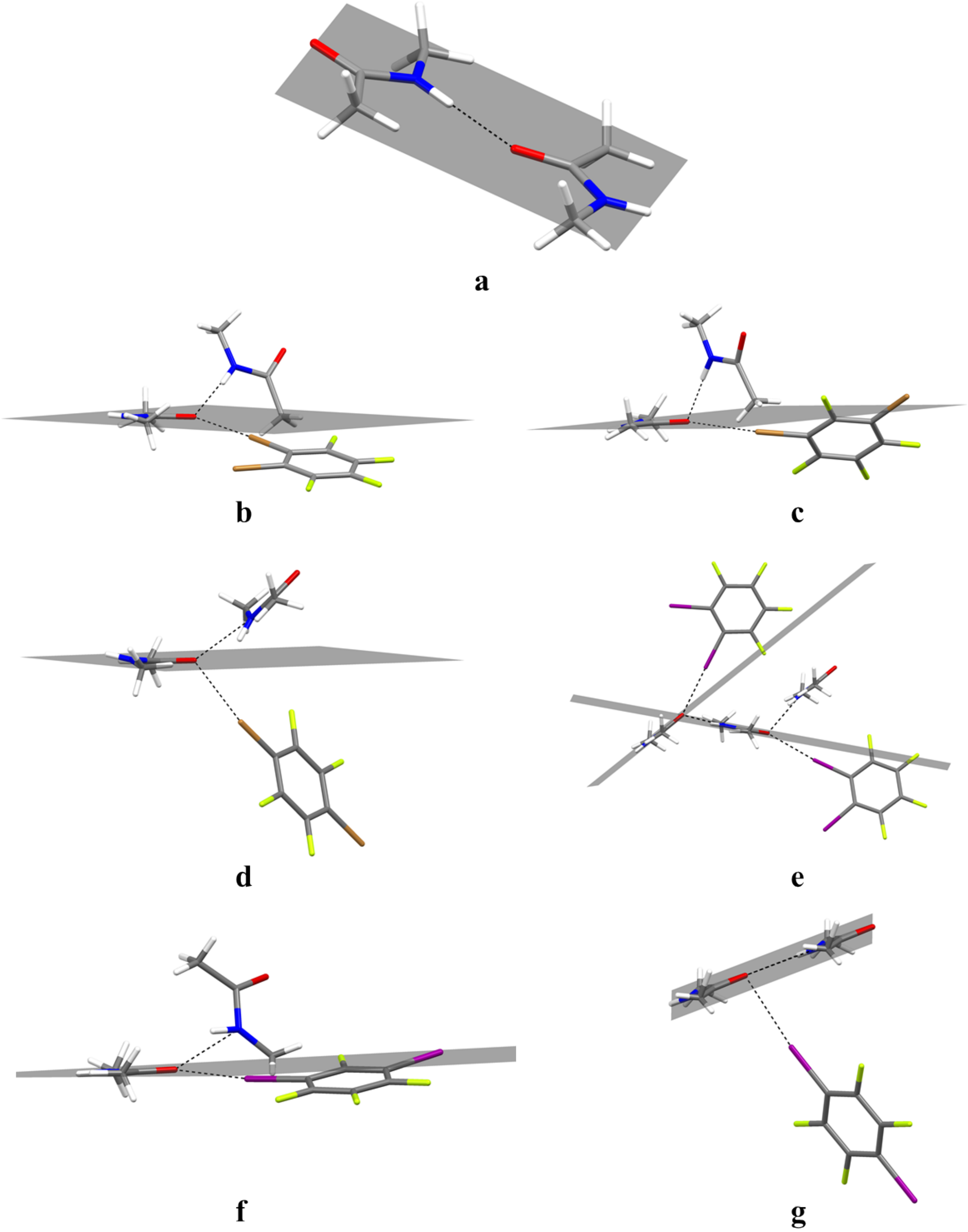

Fig. S6. N-H···O and X···O (X = I, Br) interactions in- or out-of-plane with respect to the $R_2C=O$ carbonyl group of the NMA molecule. (a) In-plane hydrogen bond in **1**. Out-of-plane Br···O and N-H···O interactions in (b) **3a**, (c) **3b**, (d) **3c**. Out-of-plane I···O and N-H···O interactions in (e) **3d**, (f) **3e**, with two planes for asymmetric NMA molecules. In-plane N-H···O HB and out-of-plane I···O XB in (g) **3f**. Planes are given in grey.

Table S4 In- and out- of R_2C=O plane angles for hydrogen and halogen bonds in **1** and **3a-f**.

Compound	∠ plane-HB (°)	∠ plane-XB (°)
**1**	0	-
**3a**	71.11	27.95
**3b**	72.57	22.03
**3c**	49.56	62.91
**3d**	52.68[a]	45.34[a]
	48.11[b]	43.28[b]
**3e**	36.19	17.59
**3f**	0	99.41

[a] angle between molecules A and C; [b] angle between molecules B and D.

S.10 References

1 R. Boese and M. Nussbaumer, in Organic Crystal Chemistry (Eds.: J. B. Garbarczyk, D. W. Jones), Oxford University Press, Oxford, 1994, pp. 20-37.

2 G. M. Sheldrick, *Acta Crystallogr. Sect. A.*, 2008, **64**, 112.

3 C. F. Macrae, I. J. Bruno, J.A. Chisholm, P. R. Edgington, P. McCabe, E. Pidcock, L. Rodriguez-Monge, R. Taylor, J. van de Streek and P. A. Wood, *J. Appl. Crystallogr.*, 2008, **41**, 466-470.

4 A. L. Spek, *Acta Crystallogr. Sect. D,* 2009, **65**, 148-155.

5 J. L. Katz and B. Post, *Acta Crystallogr.*, 1960, **13**, 624; F. Hamzaoui and F. Baert, *Acta Crystallogr.*, 1994, **C50**, 757.
